# The analysis of brain functional connectivity of post-stroke cognitive impairment patients: an fNIRS study

**DOI:** 10.3389/fnins.2023.1168773

**Published:** 2023-05-05

**Authors:** Jiahuan Zou, Yongyan Yin, Zhenfang Lin, Yulai Gong

**Affiliations:** ^1^School of Medical and Life Sciences, Chengdu University of Traditional Chinese Medicine, Chengdu,Sichuan, China; ^2^Department of Neurology, Sichuan Bayi Rehabilitation Center (Sichuan Provincial Rehabilitation Hospital), Chengdu, Sichuan, China

**Keywords:** stroke, post-stroke cognitive impairment, functional connectivity, functional near-infrared spectroscopy, assessment

## Abstract

**Background:**

Post-stroke cognitive impairment (PSCI) is a considerable risk factor for developing dementia and reoccurrence of stroke. Understanding the neural mechanisms of cognitive impairment after stroke can facilitate early identification and intervention.

**Objectives:**

Using functional near-infrared spectroscopy (fNRIS), the present study aimed to examine whether resting-state functional connectivity (FC) of brain networks differs in patients with PSCI, patients with Non-PSCI (NPSCI), and healthy controls (HCs), and whether these features could be used for clinical diagnosis of PSCI.

**Methods:**

The present study recruited 16 HCs and 32 post-stroke patients. Based on the diagnostic criteria of PSCI, post-stroke patients were divided to the PSCI or NPSCI group. All participants underwent a 6-min resting-state fNRIS test to measure the hemodynamic responses from regions of interests (ROIs) that were primarily distributed in the prefrontal, somatosensory, and motor cortices.

**Results:**

The results showed that, when compared to the HC group, the PSCI group exhibited significantly decreased interhemispheric FC and intra-right hemispheric FC. ROI analyses showed significantly decreased FC among the regions of somatosensory cortex, dorsolateral prefrontal cortex, and medial prefrontal cortex for the PSCI group than for the HC group. However, no significant difference was found in the FC between the PSCI and the NPSCI groups.

**Conclusion:**

Our findings provide evidence for compromised interhemispheric and intra-right hemispheric functional connectivity in patients with PSCI, suggesting that fNIRS is a promising approach to investigate the effects of stroke on functional connectivity of brain networks.

## Introduction

Stroke, the third leading cause of mortality globally, continues to be a significant contributor to long-term disability and cognitive impairment (Feigin et al., 2021). Stroke-induced structural damage can have widespread effects on brain function beyond the focal lesion site (Griffis et al., 2020). The risk of cognitive impairment after stroke is estimated to be five to eight times higher than in the general population (Kulesh et al., 2018). A previous study found that, despite minimal or no physical impairment, nearly half of patients with a lacunar infarct had cognitive impairment after stroke (Jacova et al., 2012). Moreover, post-stroke cognitive impairment (PSCI) is associated with an increased risk of recurrent stroke (Rostamian et al., 2014).

The assessment of cognitive function after stroke is challenging, as there is no agreed-upon gold standard for measuring PSCI. Several neuropsychological tests are commonly used, such as the Informant Questionnaire for Cognitive Decline in the Elderly (IQCODE), Oxford Cognitive Screen (OCS), Mini-Mental State Examination (MMSE), and Montreal Cognitive Assessment (MoCA) (Zhang and Bi, 2020). However, these tests may be influenced by post-stroke complications (e.g., language impairment, mood disorders) and the timing of testing (Huang et al., 2022), which could limit their validity and reliability for diagnosing and predicting PSCI. Moreover, although various biomarkers for PSCI have been proposed in recent years, such as genetic polymorphisms, inflammatory markers, growth factors, oxidative damage markers, and metabolic markers (Mijajlovic et al., 2017; Zhang and Bi, 2020), none of them have been widely accepted or validated.

Non-invasive neuroimaging techniques such as functional magnetic resonance imaging (fMRI) and functional near-infrared spectroscopy (fNIRS) have emerged as effective tools to explore the intrinsic functional organization of the human brain. Previous studies have shown a significant correlation between the hemodynamic responses measured by fNIRS and the blood oxygen level dependent (BOLD) responses obtained by fMRI, suggesting a close analogy between the two methods (Cui et al., 2011; Sato et al., 2013). However, fMRI may not be feasible for some patients due to the severity of their illness or various contraindications (Pendlebury et al., 2015). Furthermore, the noise of the MRI scanner and the discomfort of immobilization may confound the natural frequency or quality of stimulus-independent thoughts (Pinti et al., 2020), which could affect the accurate assessment of cognitive impairment. fNIRS, on the other hand, has advantages over other neuroimaging techniques, such as higher spatial resolution than EEG/MEG, higher temporal resolution than fMRI, and lower sensitivity to body movement (Huo et al., 2021). Therefore, fNIRS may be a suitable alternative for investigating the neural mechanisms of cognitive impairment related to stroke.

Functional connectivity (FC) analysis is a common method to assess brain function (Zhang et al., 2022). The human brain is a complex and dynamic system that can be modeled as a network of structural or functional connections (Niu and He, 2014). FC quantifies the temporal correlation of neurophysiological events in spatially distinct brain regions and reveals functional interactions of specific brain areas and local networks (Auer, 2008). Previous studies have shown that FC disruptions caused by lesions are strongly related to clinical impairments in various cognitive and behavioral domains (Baldassarre et al., 2016b). In the present study, we used fNIRS to examine the changes in resting-state FC among certain brain regions involved in cognitive functions in stroke patients with and without cognitive impairment and healthy controls. We aimed to improve the screening methods for PSCI risk and to elucidate the neural mechanisms of PSCI.

## Materials and methods

### Participants

Thirty-two stroke patients were recruited from Sichuan Bayi Rehabilitation Center in the present study. The inclusion criteria for stroke patients were as follows: (1) stroke that occurred 1–6 months prior to the first assessment (Dong et al., 2021), confirmed by clinical CT or MRI scanning during hospitalization; (2) age between 30 and 70 years; (3) no history of previous clinical stroke, dementia, untreated psychiatric illness, uncorrected hearing or visual impairment, aphasia or neglect; and (4) able to follow instructions and consent to participate. Also, 16 age-matched healthy participants were included as a control group. The inclusion criteria for HC were: (1) no history of neurological or psychiatric diseases; (2) normal cognitive function. All participants were right-handed and provided written informed consent in accordance with the Declaration of Helsinki. The experimental protocol was approved by the ethics committee of the Sichuan Bayi Rehabilitation Center (CKLL-20220010).

### Clinical assessment and diagnosis of PSCI

The stroke patients were divided to two groups: PSCI group and NPSCI group. The diagnostic criteria of PSCI were defined as follows: (1) the patient or a reliable informant reported cognitive impairment after stroke, or a clinician observed cognitive impairment after stroke; (2) the patient scored below the education-adjusted cutoffs on the MoCA test: ≤13 for illiterate individuals, ≤19 for those with 1–6 years of education, and ≤24 for those with 7 or more years of education (Lu et al., 2011). An experienced therapist from the Department of Rehabilitation Medicine in Sichuan Bayi Rehabilitation Center administered the Chinese version of MMSE and MoCA (Jia et al., 2021) to assess the cognitive function of all participants. Participants’ demographic and clinical characteristics are presented in Table 1.

**Table 1:** The demographic and clinical characteristics of all subjects.

Characteristics	PSCI (*n* = 16)	NPSCI (*n* = 16)	HC (*n* = 16)	*p*-value
Age (years) [median (IQR)]	62.0 (7.75)	54.5 (11.75)	54.0 (6.5)	0.060
Gender [*N* (%)]				0.135
Male	12 (75)	15 (94)	10 (63)	
Female	4 (25)	1 (6)	6 (37)	
Education (years) [median (IQR)]	7.5 (6.00)	9 (9.75)	9 (5.25)	0.447
Stroke type [*N* (%)]				0.156
Ischemic stroke	11 (69)	6 (38)	—	
Hemorrhage stroke	5 (31)	10 (62)	—	
Disease duration (days) [median (IQR)]	60 (76.75)	90 (108)	—	0.416
Hemisphere of lesion [*N* (%)]				1.000
Left	6 (38)	6 (38)	—	
Right	9 (56)	9 (56)	—	
Bilateral	1 (6)	1 (6)	—	
Lesion location [*N* (%)]				0.452
Frontoparietal lobe	4 (25)	4 (25)	—	
Brain stem	3 (19)	1 (6)	—	
Thalamus	2 (12)	0 (0)		
Basal ganglia	6 (38)	8 (50)	—	
Multiple sites	1 (6)	3 (19)		
MMSE [median (IQR)]	23.5 (5.75)	28.0 (1.00)	29.0 (1.75)	<0.001
MoCA [median (IQR)]	16 (4.25)	24 (2.00)	26 (3.00)	<0.001

PSCI, post-stroke cognitive impairment; NPSCI, post-stroke without cognitive impairment; HC, healthy controls; S, ischemic stroke; H, hemorrhagic stroke; MMSE, Mini-Mental State Examination; MoCA, Montreal Cognitive Assessment.

### Data acquisition

**Figure 1:**
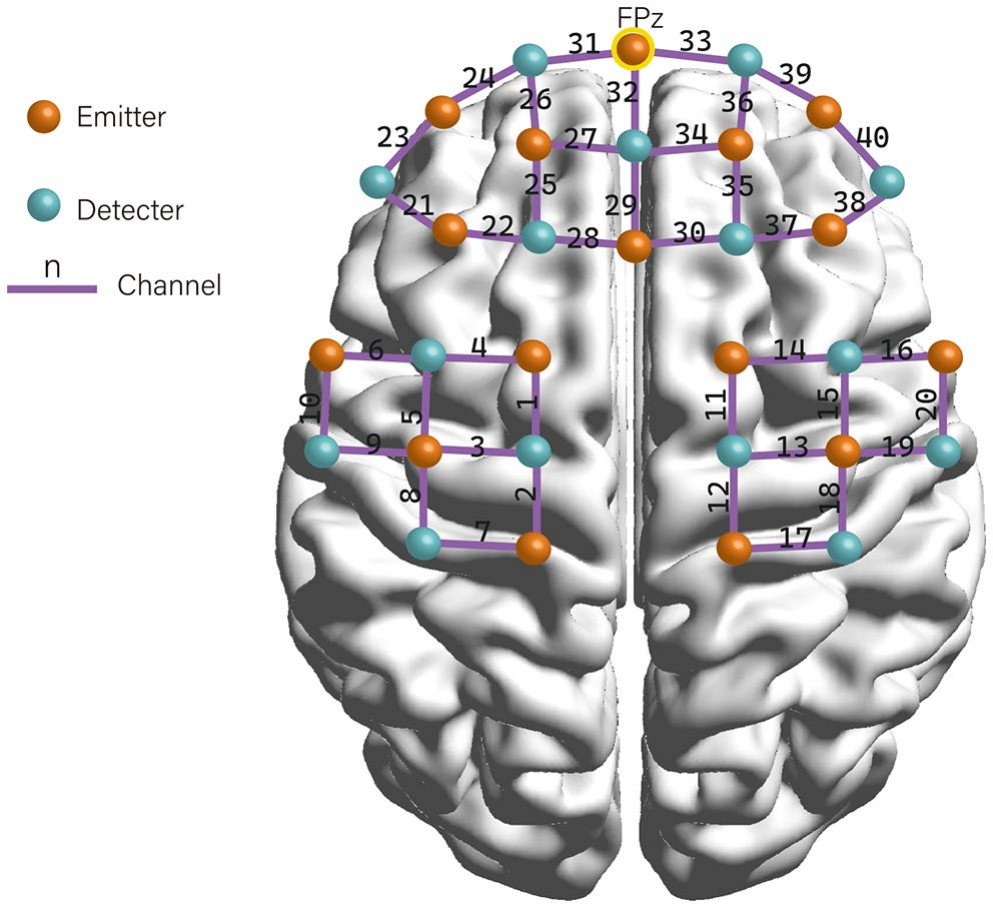
Configuration of fNIRS channels.

A multi-channel fNIRS system (NIRSport, NIRx Medical Technologies LLC) was used to measure the concentration change of oxyhemoglobin (HbO), deoxyhemoglobin (HbR), and total hemoglobin (HbT). The system recorded the absorption of near-infrared light at the wavelengths of 760 and 850 nm with a sampling frequency of 7.8 Hz. The Modified Beer–Lambert law (MBLL) (Cui et al., 2011) was used to calculate the changes in chromophore concentrations from the attenuation of light entering the head at multiple wavelengths. We placed 16 emitters and 15 detectors alternately in each location according to the international 10–20 electroencephalography (EEG) placement system, resulting in a total of 31 probes with 40 measurement channels (see Figure 1). The distance between an emitter and a detector pair was 3 cm. The area between the detector probe and emitter probe pair was defined as a “channel.” The middle optrode was placed on the FPz, and the lowest probes were positioned along the Fp1-Fp2 line. The emitters and detectors were symmetrically located in the prefrontal cortex (PFC), sensorimotor cortex (SMC), and premotor and supplementary motor cortex (PMC/SMA) regions on both hemispheres. Participants wore the fNIRS detection cap and sat in a chair. They were instructed to keep still with their eyes closed, relax their mind, and minimize their movement for at least 6 min for the resting-state recording (Guo et al., 2022).

### Data preprocessing

Preprocessing of fNIRS data was performed using Homer2 toolbox in Matlab 2013a (MathWorks Inc.). Noisy channels and unrelated time periods were manually identified and removed prior to converting the data into optical density data. Channels with motion artifacts were identified and corrected (Scholkmann et al., 2010), and removed by performing cubic spline interpolation. A 3th order Butterworth band-pass filter with cut-off frequencies of 0.01–0.1 Hz was then applied to remove artifacts, including cardiac interference (~1.3 Hz) and respiration (~0.25 Hz) (Pinti et al., 2018). Finally, the modified Beer–Lambert equation was used to convert the optical density data into HbO and HbR concentrations.

### Functional connectivity

According to the standard Brodmann brain localization, the 40 channels were divided into 13 regions of interests (ROIs), including the left/right dorsolateral PFC (DLPFC), ventrolateral PFC (VLPFC), orbital PFC (OFC), primary somatosensory cortex (S1), primary motor cortex (M1), premotor cortex and supplementary motor area (PMC/SMA), and the medial PFC (MPFC). We averaged the HbO, HbR, and HbT of all channels in each ROI and then calculated the Phase-Locking Value (PLV) as the FC value to describe the linear correlation relationship of two-time domain signals. The formula used is as follows: $PLV_{t}=\frac{1}{n}\left|\Sigma_{t=1}^{n}e^{i\left(\phi_{xt}-\phi_{yt}\right)}\right|$ , where *n* is the number of timepoints, $\phi_{xt}$ represents the phase value of the signal X at time t, and $\phi_{yt}$ is the phase value of the signal Y at time t. The value range of PLV is [0,1].

### Data analyses

Demographic and clinical differences between PSCI patients, N-PSCI patients, and healthy subjects were analyzed using SPSS Statistics 26.0. The Shapiro–Wilk test confirmed that all variables were not normally distributed. The Mann–Whitney *U*-test was used to compare disease duration and the Kruskal-Wallis H test was used to compare age, education, MMSE and MoCA. Sex, stroke types, hemisphere of lesion, and lesion location were compared using Fisher’s exact probability method.

The fNIRS data were analyzed using MATLAB (2020a, MathWorks Inc.). One-way ANOVA was performed on the hemisphere-based FC and the ROI-based FC separately. False discovery rate (FDR) was used to correct the *p*-value. Bonferroni correction was used for the multiple comparisons. A difference with *p* < 0.05 was considered statistically significant.

## Results

### Demographic and physiological characteristics

The present study recruited 33 stroke patients in total. One PSCI patient was excluded from the analysis due to his poor channel signal quality. Therefore, the final sample consisted of 16 PSCI patients, 16 NPSCI patients, and 16 HCs. Table 1 shows the demographic and clinical characteristics of the three groups. There were no significant differences in age, gender, education, stroke type, disease duration, hemisphere of lesion, and lesion location among the groups (*p* > 0.05). The PSCI group had lower MMSE and MoCA scores than the NPSCI group (*p* < 0.001). The NPSCI and HC groups did not differ significantly in MMSE and MoCA scores (*p* > 0.05, Bonferroni corrected).

### Hemisphere-based functional connectivity

**Figure 2:**
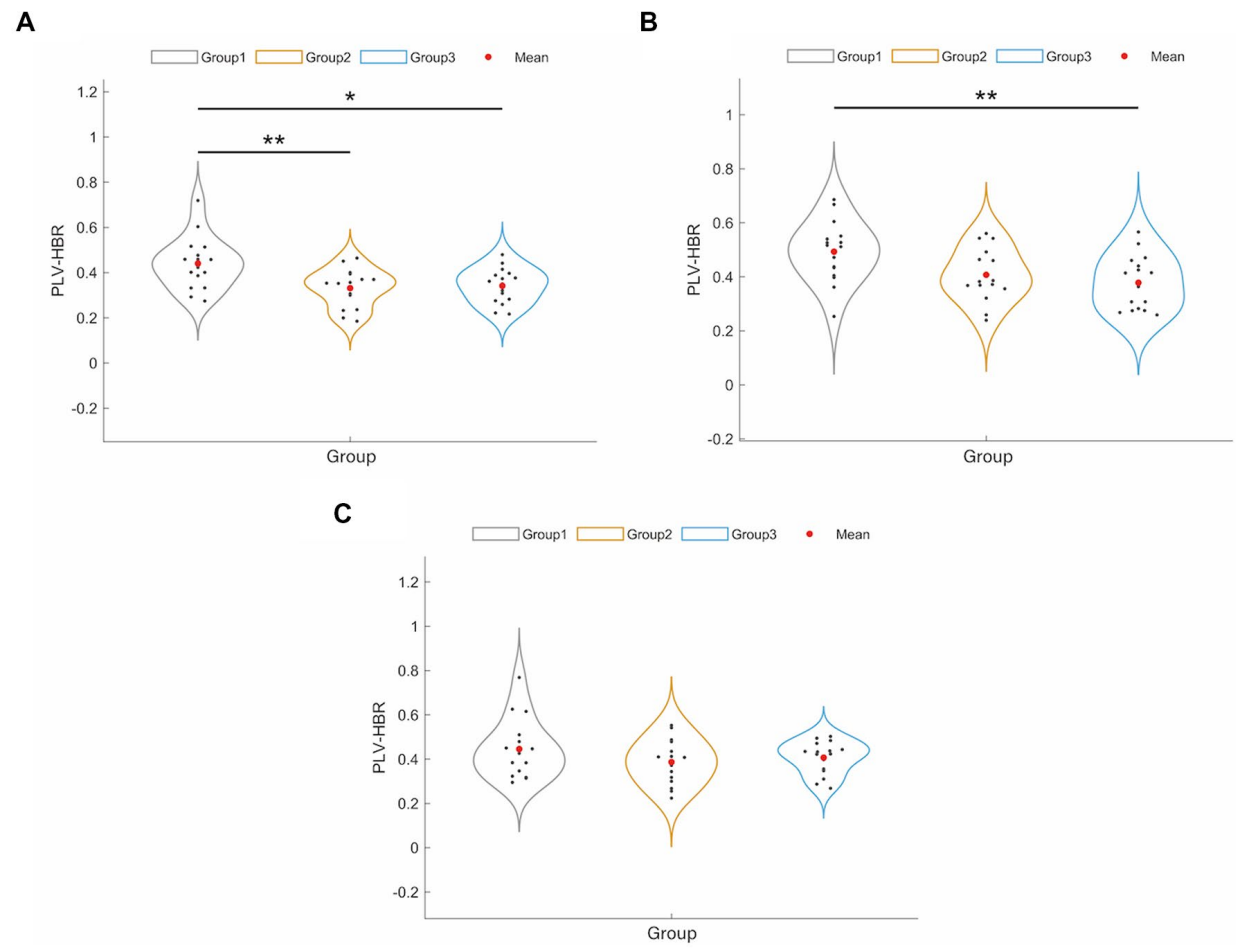
The results of significant changes of hemisphere-based FC value between HC, PSCI and NPSCI groups based on HbR. **(A)** Interhemispheric FC. **(B)** Intra-right hemispheric FC. **(C)** Intra-left hemispheric FC. Group 1: HC group; group 2: NPSCI group; group 3: PSCI group. ∗*p* < 0.05, ∗∗*p* < 0.01.

To examine the differences in connectivity patterns across hemispheres, we analyzed the FC based on three hemoglobin concentrations: HbO, HbR, and HbT. The results showed that FCs based on HbO and HbT were not significantly different across the three groups (*p* > 0.05, FDR corrected). Figure 2 shows the main changes in FC between interhemispheric and intrahemispheric regions based on HbR. The results showed that the NPSCI and PSCI groups had significantly lower interhemispheric FC than the HC group (*p* = 0.005 and *p* = 0.013, respectively, Bonferroni corrected; Figure 2A). Additionally, the PSCI group had significantly lower FC within the right hemisphere than the HC group (*p* = 0.008, Bonferroni corrected; Figure 2B). There was no significant difference in FC within the left hemisphere among the three groups (*p* > 0.05, FDR corrected; Figure 2C).

### Region-of-interest-based functional connectivity

**Figure 3:**
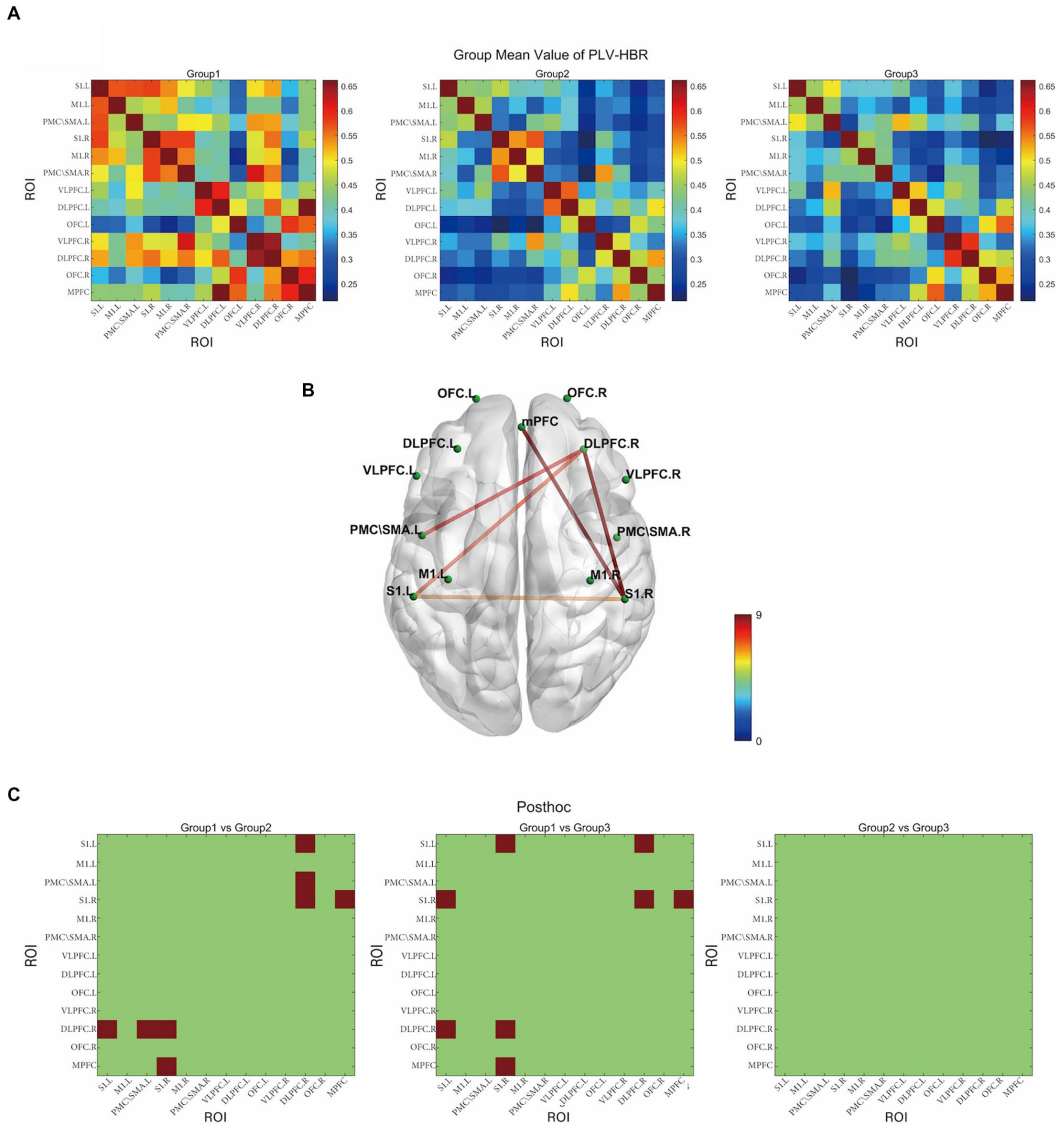
The resting-state functional connectivity in deoxyhemoglobin. **(A)** Group-averaged resting-state functional connectivity matrix diagram. **(B)** ROI-based connections with significant inter-group differences (*p* < 0.05, FDR corrected). **(C)** The results of *post-hoc* comparisons (*p* < 0.05, Bonferroni corrected). Group 1: HC group; group 2: NPSCI group; group 3: PSCI group. The color bar indicates the statistical significance threshold.

Figure 3A displays the correlation matrix maps of HbR-based FC within the HC, NPSCI, and PSCI groups. There were significant differences among the three groups in S1.L-DLPFC.R (*F* = 7.48, *p* = 0.031, FDR corrected), S1.R-DLPFC.R (*F* = 14.04, *p* = 0.001, FDR corrected), S1.R-MPFC (*F* = 9.83, *p* = 0.011, FDR corrected), PMC/SMA.L-DLPFC.R (*F* = 8.00, *p* = 0.028, FDR corrected), and PMC/SMA.L-DLPFC.R (F = 8.00, *p* = 0.028, FDR corrected; Figure 3B). *Post-hoc* Bonferroni comparisons showed that NPSCI and PSCI groups had significantly lower FC in S1.L-DLPFC, S1.R-DLPFC.R, S1.R-MPFC (*p* < 0.05) than the HC group. In addition, as shown in Figure 3C, the NPSCI group showed significantly decreased FC in PMC/SMA.L-DLPFC, and the PSCI group showed significantly decreased FC in S1.L-S1.R. However, there was no significant difference between the NPSCI and PSCI groups in any region. Similarly, the FC based on HbO and HbT were not significantly different across the three groups.

## Discussion

In the present study, we used fNIRS-based resting-state FC to investigate the changes in brain network patterns in PSCI patients compared to HCs. Our main finding was that the PSCI group showed a significant reduction of interhemispheric FC relative to HCs, which was in line with previous fMRI studies. Siegel et al. (2016) proposed the “network phenotype of stroke injury,” based on resting-state fMRI data from 100 sub-acute stroke patients, which was characterized by decreased interhemispheric FC and increased intrahemispheric FC between normally anticorrelated networks. Similar decreases in interhemispheric FC have been reported in various behavioral domains after stroke, such as attention, language, motor function, memory, and vision (Baldassarre et al., 2014, 2016a; Tang et al., 2016; Tao and Rapp, 2020; Tao et al., 2022). Our finding adds more evidence to the notion that impaired interhemispheric communication may be a key feature of stroke.

The neural mechanism underlying the reduced interhemispheric FC after stroke remains unclear. One possible explanation is that the structural connections or mechanisms that facilitate the transfer of signals between the hemispheres may be damaged or dysfunctional (Siegel et al., 2016). An animal stroke model study showed that reduced interhemispheric FC was related to decreased transcallosal manganese transport from contralesional M1 to ipsilesional SMC (van Meer et al., 2010). However, Siegel et al. (2016) found that lesion load only partially predicted the global average reduction in interhemispheric FC (*r* = 0.46), and that lesion location information did not improve the prediction. This finding suggests that interhemispheric FC disruption is a general consequence of stroke rather than a result of specific structural damage to the corpus callosum or thalamus. Tao and Rapp (2020) found that the interhemispheric FC of chronic post-stroke dysgraphia was significantly and positively correlated with spelling performance. Another study examined the predictive value of resting-state FC measured by fMRI on the third post-stroke day for the 90-day functional outcome of patients. They found that patients with better functional outcome had higher interhemispheric FC than patients with worse outcome. These findings indicate that interhemispheric FC could be a potential biomarker for the functional prognosis of stroke patients.

We also found that the PSCI group had lower FC within the right hemisphere than the HCs. There is abundant but inconsistent evidence for intra-hemispheric connectivity. Siegel et al. (2016) proposed the second stroke phenotype feature as increased FC between ipsilesional dorsal attentional network (DAN) and default mode network (DMN). They also found that this increased FC between ipsilesional functional networks was correlated with the decrease in interhemispheric FC. Similarly, increased correlations between regions within each hemisphere have been observed in monkeys after corpus callosum and anterior commissure separation (O'Reilly et al., 2013). Puig et al. (2018) suggested that structural neural adaptations may occur in more severe brain damage, with reduced interhemispheric connectivity leading to compensatory increase in ipsilateral connectivity, which may not be needed in patients with good prognosis. Tao and Rapp (2020) found that patients with chronic post-stroke dysgraphia had higher intrahemispheric connectivity in both the ipsilesional and contralesional hemispheres. However, several studies have reported lower than normal intrahemispheric FC in post-stroke aphasia (van Hees et al., 2014; Zhu et al., 2014; Nair et al., 2015). These results suggest that the effects of lesions on FC may differ across different functional networks within each hemisphere, and that the intrahemispheric FC patterns cannot be simply described as being overall higher or lower than normal. Therefore, we need to interpret this result with caution.

The analysis of ROIs showed that PSCI group had decreased FC in S1.L- S1.R, S1.L-DLPFC.R, S1.R-DLPFC.R, and S1.R-MPFC compared to HCs. Several fMRI studies have linked cognitive impairment to disruption of the default mode network (DMN) and the frontoparietal network (FPN) in stroke (Ding et al., 2014; Jiang et al., 2018; Rao et al., 2022). The DMN and FPN systems are both involved in spontaneous thought (Smallwood et al., 2012). The DMN is associated with stimulus-independent cognition and has two main hubs: the posterior cingulate cortex (PCC) and MPFC (Buckner and DiNicola, 2019). Jiang et al. (2018) found reduced connectivity within the DMN (especially the right MPFC and precuneus) in patients with acute brainstem ischemic stroke. The FPN is involved in cognitive control and mainly includes the middle frontal gyrus, dorsal anterior cingulate cortex, precuneus, caudate nucleus, and DLPFC (Andrews-Hanna, 2012). The functional integration of the FPN and overall cognitive ability have a significant positive correlation, suggesting that the strength of functional integration of the FPN and the rest of the brain is crucial for supporting superior cognitive functioning (Marek and Dosenbach, 2018). A study of dynamic functional network connectivity analysis suggested that cognitive impairment after stroke may result from a functional disconnection between the primary and FPN-centered high-order cognitive control networks. Increased FPN-centered functional network connectivity might compensate for maintaining cognitive function (Rao et al., 2022). The decreased functional connectivity of these regions may reflect functional and structural changes caused by stroke, which may impair the brain network integration and affect multiple cognitive domains.

Interestingly, the present study only found a significant difference in the connectivity derived from HbR during the resting state, and no significant results in the FC based on HbO and HbT. Nguyen et al. (2019) suggested that HbR might be more specific to the brain functional connectivity during resting state because it is more closely related to the amount of oxygen consumed by the tissues. HbO, on the other hand, is mainly related to the oxygen inflow of the brain tissue and generally considered to be the most reliable indicator for functional brain activation since it has a higher amplitude and is less affected by noise (Wolf et al., 2011). Previous studies on functional activation have shown that the BOLD signal has a stronger temporal and spatial correlation with HbR than with HbO (Huppert et al., 2006; Alderliesten et al., 2014). However, many studies have found either a slightly better correlation or a consistent correlation with HbO (Wolf et al., 2011; Duan et al., 2012; Sasai et al., 2012). Several resting-state FC studies based on fNIRS have reported that, compared to HbO, the more locally focused RSFC pattern of HbR, along with weaker RSFC strength, resulted in lower reliability (Lu et al., 2010; Zhang et al., 2010; Wolf et al., 2011), although the difference in reliability between the fNIRS parameters was small. These conflicting results indicate that more research is needed to clarify the relationship between the fNIRS parameters and their impact on RSFC.

Our work offers a new insight into the mechanisms of cognitive deficits in stroke patients, but it has some limitations. First and foremost, we recruited a relatively small number of individuals from a single institution. This ensured the quality of the data, but limited the statistical power and generalizability of the results. Moreover, the clinical characteristics of patients are quite heterogeneous depending on stroke characteristics such as type, volume, number, location (cortical or subcortical), and severity. These variables could have certain effects on characterizing neurological outcomes. In addition, we did not find a significant difference between the PSCI and NPSCI groups, which may be due to the above limitations. Therefore, a large-sample multicenter longitudinal study is needed to further investigate the pathophysiology of PSCI, which could facilitate the prevention, diagnosis and treatment of PSCI.

## Conclusion

We found reductions in interhemispheric and intra-right hemispheric FC, especially in S1.L-S1.R, S1.L-DLPFC.R, S1.R-DLPFC.R, and S1.R-MPFC, in PSCI patients. These FC changes may be involved in the pathogenic mechanism of PSCI. Therefore, resting-state fNIRS could be a promising technique to identify patients at risk of PSCI, to establish effective prevention strategies, and to guide clinical treatment.

## Data availability statement

The raw data supporting the conclusions of this article will be made available by the authors, without undue reservation.

## Ethics statement

The studies involving human participants were reviewed and approved by The Ethics Committee of the Sichuan Bayi Rehabilitation Center. The patients/participants provided their written informed consent to participate in this study.

## Author contributions

JZ, YY, and YG contributed to the conception and design of the study. JZ and YY carried out the experiments. JZ performed the data analyses and wrote the manuscript. YG and ZL provided input on grammar and rhetoric. All authors contributed to the revision of the manuscript and read and approved the submitted version.

## Conflict of interest

The authors declare that the research was conducted in the absence of any commercial or financial relationships that could be construed as a potential conflict of interest.

## Publisher’s note

All claims expressed in this article are solely those of the authors and do not necessarily represent those of their affiliated organizations, or those of the publisher, the editors and the reviewers. Any product that may be evaluated in this article, or claim that may be made by its manufacturer, is not guaranteed or endorsed by the publisher.
